# Advances in luminescence-based tracing technologies: applications and future perspectives

**DOI:** 10.1016/j.bidere.2025.100060

**Published:** 2025-10-31

**Authors:** Siyuan Sun, Tiange Wang, Yingzi Li, Hao Du

**Affiliations:** a College of Agriculture and Biotechnology, Zhejiang University, Hangzhou, 310058, China; b ZJU-Hangzhou Global Scientific and Technological Innovation Center, Zhejiang University, Hangzhou, 311215, China

**Keywords:** Tracing technologies, Fungal bioluminescent pathway, Luciferase, Green fluorescent protein, Artificial intelligence

## Abstract

Luminescence-based imaging is a fundamental tool for visualizing dynamic biological processes, primarily through bioluminescence, which generates light via enzyme-substrate reactions, and fluorescence, which requires external excitation. The recent emergence of self-sustaining systems like the fungal bioluminescence pathway (FBP) necessitates a renewed comparative analysis of these tracing technologies. Conventional tools for biological tracing, luciferases and fluorescent proteins, are limited by their dependence on external substrates or light excitation. This review systematically contrasts these methods with the self-sustaining FBP, an intrinsic metabolic circuit that enables substrate-free, autonomous bioluminescence. We conclude that integrating FBP with AI-driven protein design is the pivotal next step toward non-invasive, real-time monitoring in complex organisms, positioning FBP as a paradigm-shifting tool for future tracing technologies.

## 1 Introduction

The field of biological tracing is undergoing a transformative evolution, marked by the emergence of autonomous luminescent systems that challenge conventional imaging paradigms. While early methodologies predominantly utilized isotopic labeling, including positron emission tomography (PET) with radionuclides (e.g., ^18^F, ^11^C) and stable isotope tracking (e.g., ^15^N-labeled compounds), for metabolic and diagnostic studies [1,2], these approaches face inherent limitations in safety, handling complexity, and temporal resolution. These constraints have accelerated the adoption of luminescent techniques, which provide enhanced capabilities for real-time, non-invasive monitoring in living systems.

Among luminescent methods, bioluminescence and fluorescence imaging have become essential tools. Luciferase-based systems, exemplified by firefly luciferase, achieve exceptional sensitivity through enzyme-catalyzed light emission, enabling precise tracking of gene expression and dynamic signaling pathways [3,4]. Complementary to these, fluorescent proteins (FPs) such as green fluorescent protein (GFP) and its derivatives permit high-resolution spatial mapping of protein localization and interactions, profoundly advancing cellular and molecular imaging. Nevertheless, both techniques remain constrained by their reliance on external agents: luciferases require substrate delivery, while FPs depend on external illumination, which may introduce phototoxicity, autofluorescence, and limitations in deep-tissue penetration.

A groundbreaking shift is now underway with the development of the fungal bioluminescence pathway (FBP). Functioning as a self-sustaining metabolic pathway, the FBP enables continuous luminescence *in vivo* without exogenous substrates or light excitation [5,6]. This intrinsic autonomy, exemplified by the generation of endogenously luminous plants [7], represents a fundamental departure from conventional reporter paradigms. Yet, the translational potential of wild-type FBP from mushrooms is hampered by shortcomings in thermal stability, intensity, and spectral range. It is therefore imperative to critically synthesize how these challenges may be addressed by integrating FBP with the powerful new toolkit of artificial intelligence (AI)-driven protein optimization and *de nove* design, a synergy that is redefining the frontiers of tracing technology.

This review provides a timely and systematic analysis of the evolution of luminescent imaging, charting its course through three foundational technologies: luciferases, FPs, and FBP. We synthesize the technical advances that have established the FBP as a practical, self-sustaining system and present a roadmap for its integration with AI and synthetic biology. This convergence defines a new trajectory for the field, poised to overcome long-standing barriers and enable unprecedented capabilities in real-time, non-invasive bio-tracing.

## 2 Advances and applications in luciferase bioluminescence technology

**Fig. 1:**
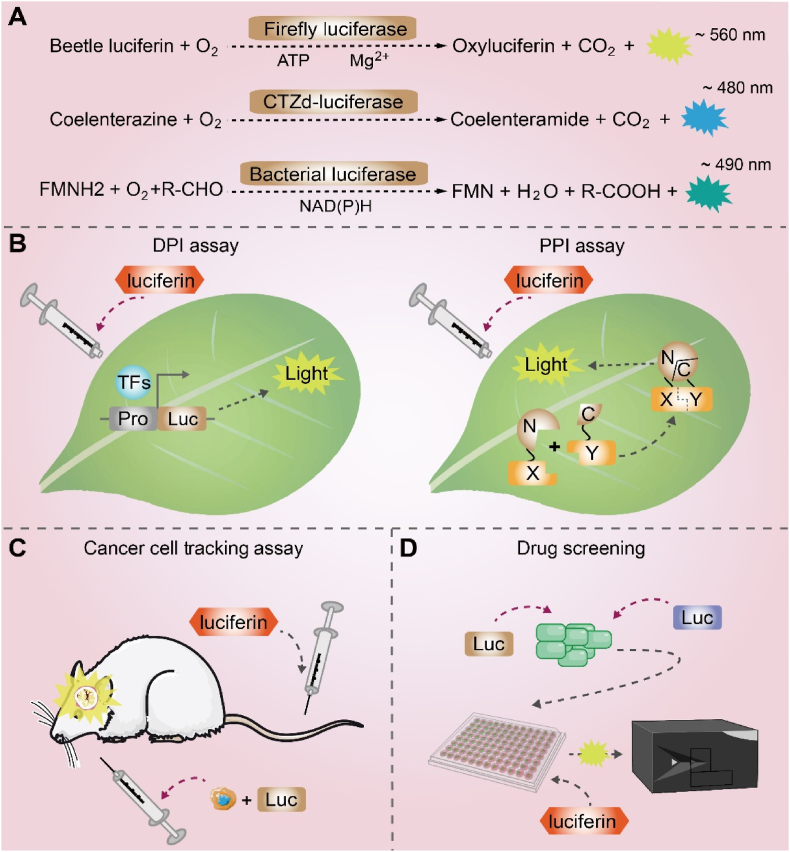
Classification and research applications of luciferases. (A) Major luciferase systems used in scientific research. Firefly luciferase oxidizes beetle luciferin in an ATP- and O_2_-dependent reaction, emitting yellow-green light (∼560 nm). Coelenterazine-dependent luciferases utilize O_2_ to oxidize coelenterazine, generating blue light (∼480 nm). Bacterial luciferase, encoded by the lux gene cluster, catalyzes the oxidation of long-chain fatty aldehydes using reduced flavin mononucleotide (FMNH_2_), producing blue-green light (∼490 nm). (B) Luciferases enable the study of DNA-protein interactions (DPI) and protein-protein interactions (PPI). (C) *In vivo* cell tracking via bioluminescence imaging. (D) High-throughput drug screening by coupling with compound libraries.

Luciferases represent a specialized group of oxidoreductases that catalyze the oxygenation of luciferin substrates to generate electronically excited oxyluciferin, which decays to its ground state via photon emission (Fig. 1A). Despite their biological ubiquity, only eleven naturally occurring luciferin-luciferase pairs have been biochemically characterized to date [8]. The firefly luciferase (FLuc) from Photinus pyralis is a 60.8 kDa enzyme that catalyzes the ATP/Mg^2+^-dependent oxidation of D-luciferin [[9], [10], [11]]. The reaction proceeds through the formation of a luciferyl-adenylate intermediate, followed by oxygenation to yield a dioxetanone [12]. The spontaneous decomposition of this dioxetanone produces excited-state oxyluciferin, which relaxes to emit characteristic yellow-green light (Fig. 1A) [13]. Owing to its high quantum yield and specificity, FLuc has become an indispensable tool for molecular imaging.

The marine environment hosts the most diverse array of bioluminescent organisms, many of which utilize coelenterazine as a common substrate despite vast phylogenetic distances [14]. This shared biochemistry involves an oxygen-dependent oxidation of coelenterazine, yielding excited-state coelenteramide and the subsequent emission of blue light, a mechanism distinct from the ATP-dependent reaction of FLuc (Fig. 1A). Among the numerous marine luciferases, three have emerged as particularly valuable reporter proteins: *Renilla luciferase* (RLuc, 34 kDa), the first characterized enzyme of this class [15]; *Gaussia luciferase* (GLuc, 19.9 kDa), which is naturally secreted due to an intrinsic signal peptide [16]; and *Metridia luciferase* (MLuc, 24 kDa), renowned for its exceptional thermostability and high secretion efficiency [17].

Quantitative performance metrics are critical for reporter selection. Firefly luciferase (FLuc) is a sensitivity benchmark, with a high quantum yield (∼0.41) and red-shifted emission ideal for deep-tissue imaging [18]. In contrast, marine luciferases typically emit blue light (∼480 nm) and have lower quantum yields (e.g., RLuc ∼0.05–0.08), facilitating multiplexed assays but resulting in greater tissue attenuation [19]. Among them, GLuc produces a higher photon flux than RLuc, making it suitable for secretion studies [20], while MLuc provides enhanced specific activity and remarkable thermal stability (>55 °C) for demanding applications [21]. Consequently, the choice of reporter is a strategic decision guided by the required sensitivity, assay format, and physiological context.

Beyond established firefly and coelenterazine-based systems, bacterial bioluminescence represents a unique, autonomous mechanism encoded by the luxCDABE(G) operon. This genetic locus, found in symbiotic bacteria such as Aliivibrio fischeri and Photorhabdus luminescens, contains all necessary components for light emission: the LuxAB luciferase and the LuxCDE enzymes for substrate synthesis (Fig. 1A) [13,22] This self-sufficient architecture eliminates the need for exogenous luciferin. However, functional expression of this prokaryotic system in eukaryotic cells remains a major challenge due to fundamental incompatibilities in substrate biochemistry and enzymatic requirements.

Luciferin-luciferase systems have become indispensable tools in biological research due to their unparalleled sensitivity and specificity. These bioluminescent reporters facilitate quantitative analyses spanning molecular to organismal levels, with three primary applications, such as gene expression profiling using promoter/3′UTR constructs and dual-reporter systems for transcriptional studies [23] (Fig. 1B), multiplexed detection of biological processes through spectrally distinct luciferases (e.g., FLuc, RLuc, GLuc) [24], as well as real-time quantification of protein-protein interactions (PPI) via split-luciferase complementation assays in plants [25] (Fig. 1B). The technology's adaptability is further evidenced by its transformative impact on *in vivo* imaging, enabling longitudinal tracking of tumor dynamics and therapeutic responses [26] (Fig. 1C). Pharmaceutical research employs these systems for high-throughput drug screening using pathway-specific reporters [27] and cytotoxicity assessments [28] (Fig. 1D).

Luciferase complementation imaging (LCI) is a bioluminescent assay for detecting and quantifying dynamic PPI *in vivo*. The technique relies on the functional reconstitution of N- and C-terminal fragments of FLuc upon interaction of their fused protein partners, generating a quantifiable luminescent signal. In contrast to irreversible methods like bimolecular fluorescence complementation (BiFC), the non-covalent nature of luciferase complementation allows for the real-time monitoring of interaction kinetics. Consequently, LCI provides a unique platform to not only detect but also quantitatively assess the stability and intensity of protein complexes [25,29].

Luciferase-based biosensors function by coupling a recognition element for a specific analyte to the enzymatic activity of a luciferase reporter. A prominent example is the MlLuc-aff biosensor, derived from a novel marine luciferase, which enables sensitive detection of HER-2 receptor overexpression in breast cancer models, highlighting its potential for clinical diagnostics [30]. The platform's versatility is further demonstrated by its adaptation for monitoring metabolic fluxes [31] and tracking plant hormone dynamics [32,33], underscoring its broad utility in both basic and applied research.

The broader application of luciferase-based imaging in deep-tissue environments remains constrained by limitations in signal intensity and emission spectra. The inherently low photon yield of conventional luciferases (e.g., FLuc, RLuc) challenges the detection of low-abundance targets, such as minute tumor cell populations. Furthermore, their predominant blue-green emission is suboptimal for *in vivo* use, as it is heavily attenuated by tissue absorption and scattering, confining detectable signals to superficial depths [34]. The development of red-shifted luciferase mutants and their engineered substrates directly addresses the spectral limitations of conventional systems. Emission at these longer wavelengths undergoes markedly reduced absorption and scattering, enabling significantly greater tissue penetration. Consequently, bioluminescence from red-shifted systems can be detected from depths exceeding several centimeters *in vivo*, a substantial improvement over conventional luciferases, whose signals are largely quenched beyond a few millimeters [35]. This enhanced penetration, facilitated by advances such as synthetic luciferin analogs (e.g., coelenterazine derivatives) and corresponding NanoLuc mutants, is critical for high-sensitivity applications in deep-seated organs and for whole-body imaging in large animal models [36].

Substrate dependency presents another major limitation, as most luciferases require exogenous luciferin administration. This requirement introduces multiple complications including substantial reagent costs, specialized equipment needs (Table 1), environmental sensitivity, potential cytotoxicity at higher concentrations, and suboptimal pharmacokinetics for longitudinal studies [37,38]. These limitations collectively reduce the technology's effectiveness for multiscale biological investigations. To overcome these barriers, future research should focus on developing enhanced luciferase systems with improved brightness, red-shifted emission spectra, and endogenous substrate production capabilities. Such innovations would markedly broaden the utility of bioluminescence imaging across diverse research applications.

**Table 1:** Characteristics of biological luminescence systems: classification and comparative advantages. λem, emission wavelength. LI, light intensity. GFP, Green fluorescent protein. EGFP, Enhanced green fluorescent protein. BFP, Blue fluorescent protein. CFP, Cyan fluorescent protein. YFP, Yellow fluorescent protein. RFP, Red fluorescent protein. DsRed, *Discosomasp* Red fluorescent protein.

Types	Categories		Parameters			Advantages	Disadvantages
			λ_em_	LI	Dependence		
Bioluminescence systems	Firefly luciferase system		∼560 nm	++	D-luciferin, ATP, Mg^2+^	(1) High sensitivity (2) Quantitative & dynamic	(1) Requires substrate addition (2) Limited multicolor options (3) Subcellular resolution (vs. FPs) (4) Signal variability (5) Low stability (6) Metabolic disturbance (7) Substrate toxicity
	Coelenterazine-dependent system		∼480 nm	+	Coelenterazine, Ca^2+^, O_2_		
	Bacterial luminescence system		∼490 nm	++	Aliphatic aldehyde, FMN, O_2_		
	NanoLuc luciferase system		∼470 nm	+++	Furimazine, O_2_		
	Fungal bioluminescence system		∼520 nm	++	Caffeic acid, NAD(P)H, ATP, O_2_	(1) No substrates addition (2) High sensitivity (3) High stability (4) Genetically encodable (5) Non-invasive detection (6) Quantitative & dynamic	(1) Metabolic disturbance (2) Large DNA fragment (3) Limited multicolor options (4) Subcellular resolution (vs. FPs)
Fluorescent proteins	Green fluorescent protein & Variants	GFP	∼509 nm	+	Excitation light, O_2_	(1) Non-invasive detection (2) Multicolor options (3) High spatial resolution (4) Low disturbance	(1) Autofluorescence interference (2) Limited tissue penetration (3) Photobleaching (4) Fluorescence quenching (5) Non-dynamic assay (6) Robust quantification: challenging (7) UV damage (BFPs, CFPs)
		EGFP	∼507 nm	++			
		BFP/CFP /YFP	∼440/475 /527 nm	+/+ /++			
	Red fluorescent protein & Variants	DsRed	∼583 nm	+++			
		mCherry	∼610 nm	+			
		tdTomato	∼581 nm	+++			

## 3 Fluorescent protein-based tracing technology

**Fig. 2:**
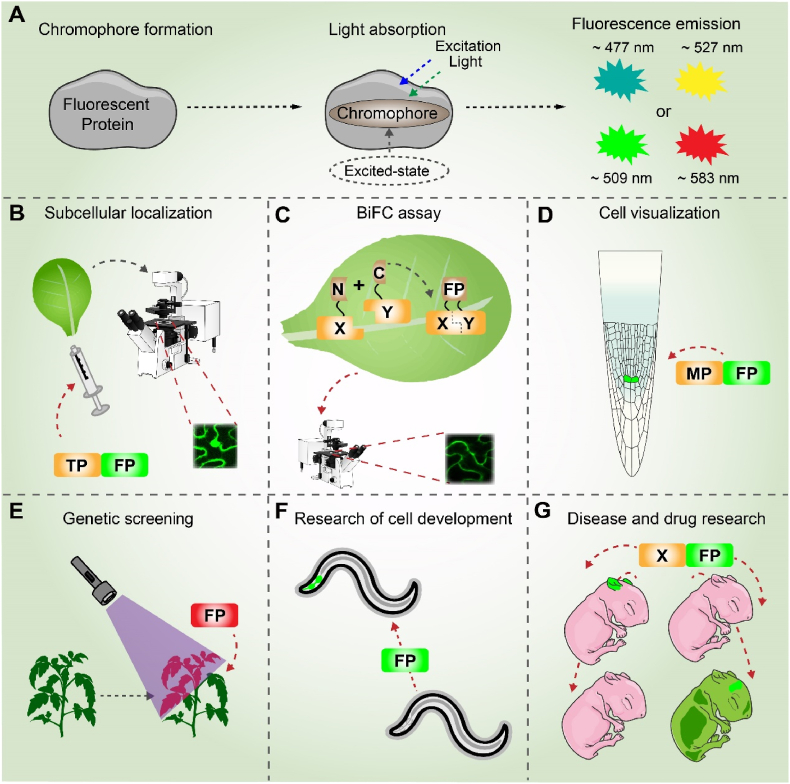
Luminescence mechanism and research applications of FPs. (A) FPs emit light via chromophore structural transformations and energy transitions. The chromophore forms through autocatalytic cyclization, dehydration, and oxidation of internal amino acids. Upon light absorption at specific wavelengths, chromophore electrons transition from the ground to excited state, followed by fluorescence emission via radiative decay. (B–G) Research applications of FPs include (B) Subcellular localization by fusion with target proteins (TPs). (C) Bimolecular fluorescence complementation (BiFC) assays using split-complementation. (D) Monitoring cell-specific expression patterns. (E) Labeling exogenous gene transfer. (F) Tracing cellular dynamics during development. (G) Tracking disease progression in cell transplantation studies. Abbreviations: FP, fluorescent protein; MP, marker protein; TP, target protein.

GFP, since its discovery in *Aequorea victoria* and subsequent establishment as a genetic reporter, has become an indispensable tool for live-cell imaging [39,40]Its function originates from a unique p-hydroxybenzylidene-imidazolinone chromophore that forms autocatalytically within a protective β-barrel scaffold (Fig. 2A). GFP's fluorescent properties stem from its chromophore, which displays dual absorption peaks at ∼395 nm and ∼475 nm, corresponding to its protonated and deprotonated states, respectively [41]. The interconversion between these states is facilitated by a proton transfer cascade through a hydrogen-bond network involving residues Glu 222 and Ser 205 [42]. The slow relaxation kinetics of this intrinsic photocycle confer the exceptional photostability that is fundamental to GFP's utility as a robust fluorescent reporter *in vivo*.

The engineering of a diverse palette of FPs, from blue (BFP) and cyan (CFP) to yellow (YFP) and red (DsRed) variants, has provided a versatile toolkit for live-cell imaging [43,44]These genetically encoded reporters have been particularly transformative through the development of Fluorescence resonance energy transfer (FRET)-based biosensors, which convert molecular interactions, such as protein dynamics, into quantifiable fluorescence signals [45] (Fig. 2A–C).

This technology has enabled groundbreaking applications across biological kingdoms. In plant systems, FPs have been instrumental in tracking transgene expression [46] (Fig. 2D and E), assessing CRISPR/Cas9 editing efficiency [47], and visualizing fundamental processes like root development [48] and the inter-organellar transduction of stress signals [49]. Concurrently, in mammalian systems, FPs permit the single-cell resolution analysis of complex processes like organogenesis and have become indispensable in disease modeling and drug discovery [50,51] (Fig. 2F and G). However, the application of FPs in plant systems presents unique challenges. High autofluorescence from chlorophyll and cell walls in the green spectrum creates significant background noise, often obscuring signals from common reporters like GFP. Furthermore, the unique physiology of plant cells, including heterogeneous oxygen concentrations and a dominant vacuolar compartment that can perturb protein folding and stability, complicates FP maturation and accurate interpretation of localization data. These constraints necessitate careful optimization of FP selection and experimental design, diverging from standard animal cell protocols.

FPs have revolutionized the analysis of biomolecular interactions, enabling sophisticated techniques such as multicolor FRET, fluorescence lifetime imaging, and BiFC [[52], [53], [54], [55]]. As genetically encoded biosensors, they allow non-invasive, spatiotemporally precise monitoring of a vast array of cellular analytes, including ions, signaling molecules, metabolites, and phytohormones, hen fused to proteins or incorporated into molecular switches. Their translational utility is further demonstrated by applications such as GFP-reporter viruses for antiviral screening [56], cementing their role as indispensable tools that bridge fundamental research and biomedical applications.

Despite their utility, FP-based systems face significant constraints for quantitative applications. Key challenges include variable expression levels and inconsistent maturation kinetics, which complicate signal normalization and accurate quantification across samples. Furthermore, their dependence on fluorescence microscopy imposes substantial infrastructure requirements and specialized expertise. These practical issues are compounded by intrinsic photophysical limitations that can undermine data reliability. The most critical limitations stem from the inherent properties of FPs themselves. **Photobleaching** irreversibly diminishes signal over time; for instance, EGFP can exhibit a photobleaching half-life of less than 60 s under standard imaging conditions, precluding long-term longitudinal studies. **Oligomerization** tendencies, such as the tetramerization of wild-type DsRed, can artificially cluster fusion proteins and perturb native biological interactions [57]. Furthermore, **environmental sensitivity** leads to fluorescence quenching by factors like low pH, which can reduce the quantum yield of GFP-based probes by over 50 % in acidic compartments [58]. Finally, robust quantification with FPs remains challenging due to expression variability, maturation kinetics, and photobleaching. These inherent photophysical limitations ultimately restrict their utility for precise measurements in physiologically relevant environments (Table 1).

FP systems face inherent biological limitations, including high-energy excitation light that induces phototoxicity and photobleaching, thereby restricting longitudinal studies (Table 1) [59]. The development of near-infrared FPs (NIR FPs; e.g., iRFP/miRFPs), which are excited within the tissue transparency window (650–900 nm), represents a significant advance by minimizing light scattering, autofluorescence, and photodamage [60]. While FPs remain indispensable, these limitations hinder their use in scalable and clinical contexts, driving current research to engineer FPs with enhanced biocompatibility and to develop complementary, non-fluorescent labeling strategies.

## 4 Biochemistry and pathway mechanism of the FBP

Fungal bioluminescent systems, particularly the caffeic acid metabolic pathway known as the FBP, represent a unique and evolutionarily conserved mechanism for biological tracing, distinct from conventional luciferase- and fluorescent protein-based technologies. This pathway, identified across diverse fungal lineages such as *Armillaria*, *Mycenoid*, *Omphalotus*, and *Lucentipes*, exhibits remarkable biochemical consistency. Its presence in edible species like *Armillaria mellea* further suggests favorable biosafety profiles [[61], [62], [63]]. Early foundational studies established the NAD(P)H-dependent nature of fungal bioluminescence [64] and revealed shared luciferin-luciferase components across species [65].

**Fig. 3:**
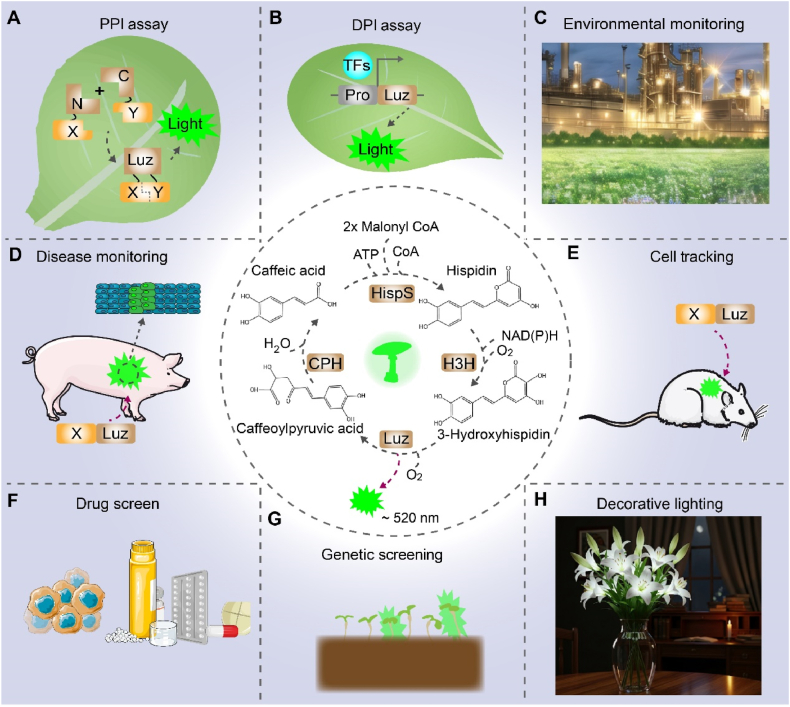
Mechanism and applications of fungal bioluminescence systems. The fungal bioluminescence pathway (FBP) generates green light emission (∼520 nm) through enzymatic oxidation of endogenous substrates (e.g., caffeic acid and hispidin). This system, which is currently being developed as a research tool, involves four key enzymes: hispidin synthase (HispS), hispidin-3-hydroxylase (H3H), luciferase (Luz), and caffeyl pyruvate hydrolase (CPH), with CoA and NAD(P)H as essential cofactors. Potential research applications include: (A) Detecting PPI with a split-protein complementation assay. (B) Profiling transcriptional regulation through DPI assay. (C) A biosensing assay for the continuous monitoring of environmental pollutants. **(D)** Detection of tissue lesions via gene expression signatures. **(E)** Tracking of cancer cell proliferation and metastasis. **(F)** High-throughput drug screening. **(G)** Identification of transgenic plants. **(H)** Engineering of glowing ornamental plants for decorative bioluminescence. Abbreviations: TF, transcription factor. Pro, promoter. DPI, DNA-protein interactions. PPI, protein-protein interactions.

The complete FBP was fully elucidated in *Neonothopanus nambi*, revealing a four-enzyme cycle that constitutes a self-sustaining metabolic pathway [6]. The cycle initiates with Hispidin synthase (HispS) converting caffeic acid to hispidin, which is subsequently hydroxylated by H3H to form the actual luciferin, 3-hydroxyhispidin. Oxidation of this luciferin by luciferase yields green light emission (*λ*_max_ = 520–530 nm) and caffeylpyruvate. The pathway is completed by CPH, which regenerates caffeic acid from caffeylpyruvate (Fig. 3). This elegant cycle requires multiple cofactors, including CoA, malonyl-CoA, NAD(P)H, ATP, and oxygen, for continuous operation. The self-sustaining nature of this pathway, coupled with its evolutionary conservation across fungal species and demonstrated biosafety in edible varieties, positions fungal bioluminescence as a promising alternative for biological tracing applications where conventional systems may be limited. The system's metabolic recycling capability enables continuous light production, offering distinct advantages for long-term monitoring applications.

A critical feature of the FBP is its metabolic autonomy in heterologous systems. The pathway utilizes caffeic acid, a metabolite native to the plant phenylpropanoid pathway that is also endogenously produced in yeast and mammalian cells via tyrosine metabolism [66]. This convergence enables substrate-independent bioluminescence upon heterologous expression of the FBP enzymes. Kotlobay et al. successfully demonstrated this principle by reconstituting the core FBP enzymes in *Pichia pastoris*, human cells, and *Xenopus laevis* embryos, achieving autonomous luminescence without exogenous substrate. The system has been notably successful in engineering autonomously bioluminescent plants, which exhibit at least an order of magnitude greater brightness than those using bacterial bioluminescence systems [67]. Subsequent research has focused on optimizing metabolic flux through the FBP via improved energy and cofactor management, with metabolic engineering strategies being employed to overcome intrinsic substrate limitations and further enhance luminescent output in plants [68,69].

## 5 Applications of FBP in tracing technologies

The FBP represents a transformative advance in biological reporting technology, establishing itself as a versatile platform for diverse research applications. The system's autonomy, derived from its endogenous caffeic acid metabolism, permits real-time observation of key processes without the need for external substrates. This capability is exemplified by its use in monitoring protein-protein interactions through split-luciferase complementation (Fig. 3A) and in probing the dynamics of gene expression and transcriptional regulation (Fig. 3B). This autonomy, combined with broad dynamic range and high-throughput compatibility, makes FBP particularly valuable for plant synthetic biology [70,71]. The translational potential of the FBP system was demonstrated by Sun et al. who developed a FBP-based reporter exhibiting superior reliability, sensitivity over existing systems including FLuc, and operational simplicity across multiple plant species [5]. The favorable biosafety profile of FBP, supported by the edibility of some native bioluminescent fungi and the low toxicity of its endogenous precursor caffeic acid, suggests a reduced biosafety risk compared to conventional luciferase systems. Consistent with this, the minimal cellular toxicity and high metabolic stability of FBP components (Table 1) enabled the generation of stable transgenic lines suitable for quantitative longitudinal analysis.

The modular architecture of the FBP system enables its adaptation for diverse environmental monitoring applications, such as pollutant detection and indoor formaldehyde monitoring (Fig. 3C), though further improvements in sensitivity and operational stability are needed for broad implementation. A notable technical advance is the recent development of a dual-responsive biosensor capable of non-invasively visualizing salicylic acid and jasmonic acid signaling dynamics in response to biotic stress. This system integrates fungal-derived bioluminescence genes with stress-responsive promoters, creating a functionally validated tool for studying plant immune responses [72]. The non-invasive nature of FBP further supports continuous monitoring of disease progression (Fig. 3D), highlighting its utility in biomedical research. Moreover, the real-time luminescence output enables high-throughput cell population tracing (Fig. 3E) and drug screening to precisely quantify cellular responses to pharmacological compounds (Fig. 3F).

Recent advances in enzyme engineering have begun to address these limitations, leading to improvements in FBP catalytic efficiency and stability [73]. These refinements are expanding its utility in quantitative, dynamic biological investigations (Fig. 3) [74]. As a sustainable biotechnology, FBP's nontoxic, autonomous nature aligns with the demand for eco-friendly solutions, with potential applications ranging from commercial autoluminescent plants to real-time environmental biosensors [74]. The NAD(P)H-dependent nature of the pathway further allows it to function as a metabolic sensor, capable of detecting various organic and inorganic toxicants, as evidenced in native luminescent fungi [75,76].

The implementation of FBP technology effectively addresses persistent challenges in plant high-throughput screening (HTS), where physical constraints from cell walls and complex growth requirements have limited conventional approaches. While pigment-based systems like RUBY represented an advance for plant studies [77], they remain constrained by sensitivity limitations and background interference. In contrast, FBP's substrate independence, compatibility with intact plants, and seamless integration with automated imaging platforms have significantly enhanced genetic screening efficiency (Fig. 3G). Recent studies have demonstrated FBP's efficacy in quantitative metabolic network analysis, highlighting its potential for robust HTS when integrated with established plant biotechnologies such as protoplast assays and cell culture systems [70].

**Fig. 4:**
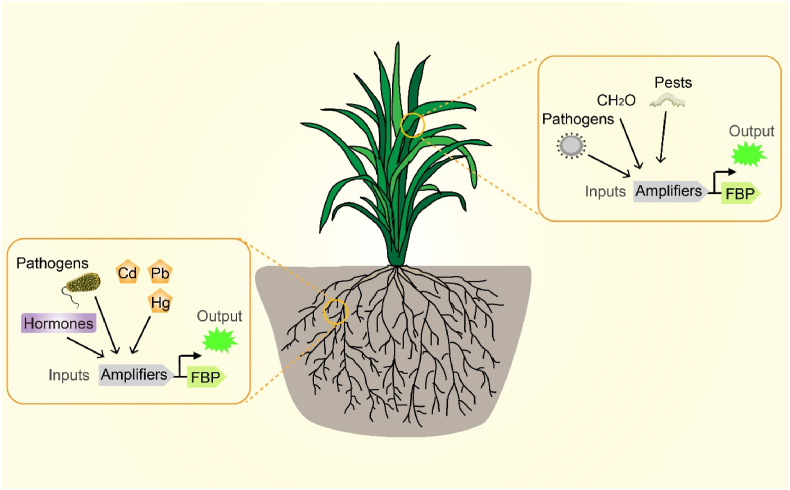
Design of an FBP-based plant biosensor. The FBP was engineered into plants through genetic circuits incorporating effecter-responsive circuits, coupled with signal amplification systems for enhanced detection sensitivity. This biosensor enables real-time monitoring of environmental stressors, including pathogens and heavy metals (Cd, Pb, Hg). Abbreviations: FBP, fungal bioluminescence pathway; Cd, cadmium; Pb, lead; Hg, mercury.

When engineered with effector-responsive regulatory elements, the FBP constitutes a transformative biosensing platform. Its superior cross-species compatibility compared to bacterial systems makes it ideal for terrestrial applications, including agricultural monitoring, pathogen surveillance, and tracking of disease progression in plants. With further optimization of its sensitivity and operational stability, FBP-based systems hold exceptional promise for high-sensitivity environmental monitoring, indoor air quality assessment, and precision integrated pest management (Fig. 4). The platform's versatility across plant and animal models solidifies its status as a next-generation tracing technology with significant potential to advance both fundamental research and applied biotechnology, including the development of autoluminescent plants.

## 6 Limitations and challenges of FBP

Despite its considerable promise as an autonomous reporting system, the FBP faces several technical challenges that must be resolved for broad implementation. A primary constraint is its limited photon output. While FBP-based systems exceed the brightness of bacterial bioluminescence, their signal intensity remains substantially lower than that of optimized exogenous substrate systems such as NanoLuc, particularly in contexts requiring high signal-to-background ratios or deep-tissue penetration.

Furthermore, the coordinated expression of four enzymatic components imposes a significant metabolic burden on host cells, potentially perturbing native physiology and transgene stability. The pathway's light production is also intrinsically linked to central metabolism, fluctuating with cellular pools of caffeic acid and NAD(P)H rather than providing a constant baseline [69]. This metabolic dependency complicates its use in quantitative assays requiring stable signals. Current research focuses on mitigating these limitations through enzyme engineering to improve catalytic efficiency, refactoring genetic constructs to reduce metabolic load, and employing synthetic regulatory circuits to decouple pathway activity from endogenous metabolic noise.

Current research is focused on addressing these limitations through protein engineering to enhance catalytic efficiency and stability, refactoring genetic constructs to minimize metabolic load, and employing synthetic regulatory circuits to decouple pathway activity from endogenous metabolic noise. While the FBP system represents a transformative platform for substrate-free imaging and sensing, these critical challenges in brightness, metabolic integration, and output stability must be overcome to realize its full potential in both fundamental and applied settings.

## 7 Future perspectives

Bioluminescent and fluorescent technologies represent cornerstone methodologies in biological research, enabling real-time analysis of gene expression, protein interactions, and dynamic processes in living systems. Recent advances in protein engineering, artificial intelligence (AI), and metabolic optimization are poised to overcome longstanding limitations in sensitivity, spectral range, and tissue penetration, thereby expanding the scope of these technologies for both fundamental and translational applications.

(1) **Engineered luciferases with enhanced performance**

The demand for bioluminescent reporters with superior intensity and reliability has driven the rational engineering of luciferases. Substantial progress has been made in engineering luciferases with improved brightness and stability. The development of NanoLuc luciferase from *Oplophorus gracilirostris* marked a breakthrough, providing a ∼150-fold signal enhancement over traditional systems (FLuc/RLuc) while maintaining superior physical stability [78,79]. Its successful adaptation for protein-protein interaction assays via rational splitting strategies further demonstrates its versatility [80,81]. Similarly, chimeric luciferases such as PpyLit, which combines functional domains from *Photinus pyralis* and *Luciola italica*, exhibit enhanced catalytic efficiency and photon output [82]. These engineered luciferases significantly improve signal-to-noise ratios and expand quantitative imaging capabilities.

(2) **Next-generation FPs**

Parallel to the engineering of luciferases, the fluorescent protein toolkit has undergone a similar revolution, being substantially enriched by novel variants that overcome historical constraints of photobleaching and low brightness. Proteins such as mNeonGreen [83], the exceptionally photostable StayGold [84], and the engineered pair mClover3/mRuby 3 [85] offer exceptional performance, enabling prolonged and sensitive imaging. By directly addressing critical limitations in temporal resolution, these next-generation FPs support more reliable long-term studies of cellular dynamics.

(3) **AI-driven protein design and directed evolution**

AI has revolutionized the design of novel imaging proteins. The Baker laboratory employed deep learning methods to design LuxSit, a fully *de novo* luciferase (13.9 kDa) exhibiting exceptional thermal stability and strong substrate specificity (>50-fold preference for diphenylterazine) [86]. Subsequent optimization yielded the Neolux series, even more compact (13.7 kDa) and catalytically efficient enzymes capable of forming functional FRET pairs for multiparametric imaging *in vivo* [87]. In parallel, computational design facilitated the creation of a fluorescence-activating transmembrane protein that exhibits>1600-fold activation upon ligand binding, achieved through AlphaFold2-guided structural engineering [88]. These approaches are complemented by directed evolution, which continues to refine existing luciferases and FPs for enhanced performance in complex biological environments.

(4) Expanding the spectral palette for deep-tissue imaging

Significant efforts have focused on developing long-wavelength emitters to enhance tissue penetration. Bioluminescent systems have been engineered through both luciferase modification and substrate derivatization. For example, a red-shifted NanoLuc variant developed via C-8 modification of coelenterazine offers brighter emission and improved deep-tissue performance [36]. The Nano-lanternX system further expanded this palette, utilizing bioluminescence resonance energy transfer to generate five spectrally distinct luciferases applicable across diverse organisms [33]. In the fluorescent protein realm, near-infrared variants have been engineered through both natural protein optimization and synthetic chemistry. A notable example includes a 772 nm emitter created by incorporating a merocyanine retinal chromophore into Archaerhodopsin-3 [89], while synthetic dyes such as styrene oxazolone derivatives provide large Stokes shiftsand excellent performance in tumor and brain imaging [90]. While near-infrared FPs generally offer higher photon output and simpler instrumentation, bioluminescent probes excel in contexts requiring minimal autofluorescence and no photobleaching. The choice between these modalities depends on specific experimental needs regarding sensitivity, temporal resolution, and tissue depth.

(5) **Optimization strategies for FBP**

**Fig. 5:**
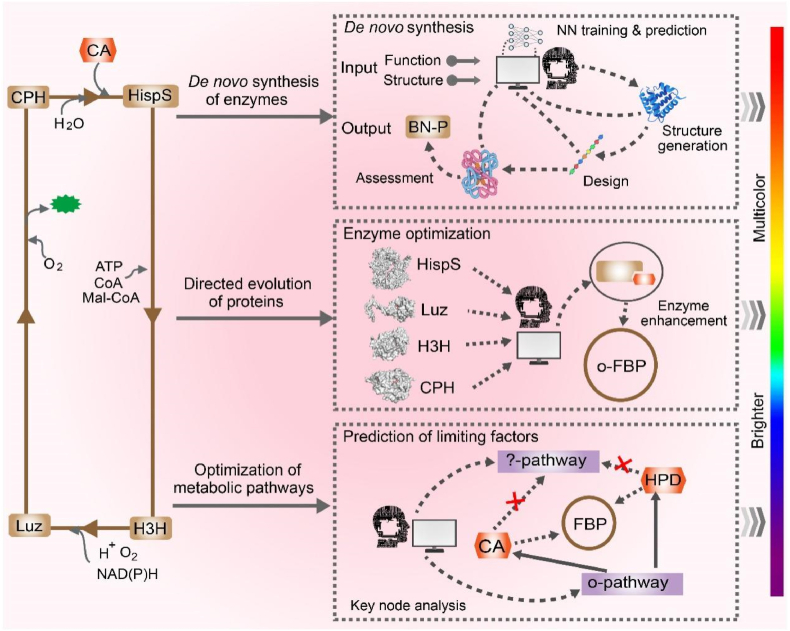
Optimization strategies for the FBP system. Three principal approaches are employed to enhance FBP performance: (1) *De novo* design of novel luminescent enzymes using AI-based tools for customized applications; (2) Improvement of catalytic efficiency through directed evolution of core FBP enzymes; and (3) Systematic optimization of the FBP metabolic circuit to augment substrate availability (CA, HP) and reaction kinetics. Abbreviations: oFBP, optimized FBP; CA, caffeic acid; HP, hispidin.

Building on these engineered reporters, the emerging FBP represents a paradigm shift, however, realizing its potential requires systematic optimization. To achieve this, efforts are focused on multiple fronts: enhancing heterologous expression in plants, animals, and microbes; optimizing regulatory elements and protein stability; and expanding spectral properties. Three principal strategies are being employed (Fig. 5): (i) AI-driven *de novo* design of luciferases with customized properties; (ii) directed evolution of core FBP enzymes to improve catalytic efficiency and stability; and (iii) metabolic engineering to optimize substrate availability and pathway kinetics. These integrated approaches are essential for broadening FBP's utility in research and real-world applications.

## 8 Conclusions

Luminescence-based tracing is now a fundamental methodology for the real-time analysis of biological processes. The integration of these tools with plant synthetic biology, genome engineering, and AI-assisted biodesign, exemplified by platforms like PlantGPT, is unlocking unprecedented opportunities in basic and translational research [[91], [92], [93]]. This synergistic convergence of protein engineering, computational design, and metabolic optimization is paving the way for a new generation of autonomous bioluminescent systems. Ultimately, the future of biological tracing lies in creating intelligent, adaptive systems that provide exceptional clarity and precision. These self-sustaining technologies are poised to transcend the laboratory, enabling transformative applications in real-time environmental monitoring and sustainable lighting to address pressing ecological and societal challenges.

## 9 Image usage statement

The biological illustrations featured in Fig. 1, Fig. 2, Fig. 3, Fig. 4, Fig. 5, including representations of rat, pig, bacterial, cancerous cell, Arabidopsis, microscope, nematode, rat embryo, tobacco, and protein models, were sourced from Servier, Frédéric Bouché, and DBCLS through Bioicons.com. These images are licensed under Creative Commons Attribution 3.0 (CC BY 3.0) and 4.0 (CC BY 4.0) licenses, with select public domain exclusions as specified on the platform. The author confirms compliance with all licensing requirements, including proper attribution, and acknowledges modifications made to certain images regarding coloration and illustrative details. Complete license terms are available at https://creativecommons.org/licenses/by/3.0/and https://creativecommons.org/licenses/by/4.0/. This statement affirms adherence to all copyright regulations governing the use of these visual assets in academic publication.

## Author contributions

H.D. conceptualized the manuscript outline. H.D., S.S., T.W., and Y.L.contributed to the writing. S.S. and T.W. created the figures. All authors participated in discussions and revised the manuscript. S.S. and T.W. contributed equally to this work.

## Declaration of competing interest

The authors declare that they have no known competing financial interests or personal relationships that could have appeared to influence the work reported in this paper.
